# Lower frequency routine surveillance endomyocardial biopsies after heart transplantation

**DOI:** 10.1371/journal.pone.0182880

**Published:** 2017-08-25

**Authors:** Ludwig T. Weckbach, Ulrich Maurer, Rene Schramm, Bruno C. Huber, Korbinian Lackermair, Max Weiss, Bruno Meiser, Christian Hagl, Steffen Massberg, Sandra Eifert, Ulrich Grabmaier

**Affiliations:** 1 Department of Medicine I, Ludwig-Maximilians-University, Campus Grosshadern, Munich, Germany; 2 Department of Cardiac Surgery, Ludwig-Maximilians-University, Munich, Germany; 3 Transplantation Center, Ludwig-Maximilians-University, Munich, Germany; 4 Institute of Pathology, Ludwig-Maximilians-University, Munich, Germany; 5 DZHK (German Centre for Cardiovascular Research), Partner Site Munich, Germany; Universita degli Studi di Bologna, ITALY

## Abstract

In heart transplantation (HTx) patients, routine surveillance endomyocardial biopsies (rsEMB) are recommended for the detection of early cardiac allograft rejection. However, there is no consensus on the optimal frequency of rsEMB. Frequent rsEMB have shown a low diagnostic yield in the new era of potent immunosuppressive regimen. Efficacy and safety of lower frequency rsEMB have not been investigated so far. In this retrospective, single centre, observational study we evaluated 282 patients transplanted between 2004 and 2014. 218 of these patients were investigated by rsEMB and symptom-triggered EMB (stEMB). We evaluated EMB results, complications, risk factors for rejection, survival 1 and 5 years as well as incidence of cardiac allograft vasculopathy (CAV) 3 years after HTx. A mean of 7.1 ± 2.5 rsEMB were conducted per patient within the first year after HTx identifying 7 patients with asymptomatic and 9 patients with symptomatic acute rejection requiring glucocorticoide pulse therapy. Despite this relatively low frequency of rsEMB, only 6 unscheduled stEMB were required in the first year after HTx leading to 2 additional treatments. In 6 deaths among all 282 patients (2.1%), acute rejection could not be ruled out as a potential underlying cause. Overall survival at 1 year was 78.7% and 5-year survival was 74%. Incidence of CAV was 17% at 3-year follow-up. Morbidity and mortality of lower frequency rsEMB are comparable with data from the International Society for Heart and Lung Transplantation (ISHLT) registry. Consensus is needed on the optimal frequency of EMB.

## Introduction

Routine surveillance endomyocardial biopsies (rsEMB) are considered to be important to detect acute rejection after heart transplantation (HTx) [1]. The working formulation for acute cellular rejections, originally defined in 1990, has been revised in 2004. Here, rejections were categorized in grade 0R for no rejection (no change from previous classification), grade 1R for mild rejection (summarizing grade 1A, 1B and 2 of the former classification), grade 2R for moderate rejection (reflecting former grade 3A) and grade 3R for severe rejection (summarizing former grade 3B and 4) [2]. According to the International Society for Heart and Lung Transplantation (ISHLT) guidelines, symptomatic grade 1R rejections or rejections ≥ grade 2R require treatment with pulsed glucocorticoids (GC), whereas asymptomatic grade 1R rejections do not require treatment [3].

The incidence of early acute cellular rejection after heart transplantation decreased significantly during the last two decades. In 1998, Kobashigawa et al. observed ≥ grade 3A rejections in 45–52.9% after a 6-month follow-up in their randomized study on mycophenolate mofetil (MMF) [4]. Ten years later, Hamour et al. revealed a cumulative incidence of ≥ grade 3A rejections at 1-year follow-up of only 20.7–29.3% [5].

Likewise, in the ISHLT registry database, the authors report a decline of rejections requiring treatment from 23% (transplanted between 2004 and 2006) to 13% (transplanted between 2010 and 2011) [6]. Overall, the decline in clinically relevant rejections was attributed foremost to more efficient immunosuppressive regimen and led to the discussion, whether the high frequency of rsEMB practiced in many centres is still adequate [5–7]. For example, Hamour et al. revealed a diagnostic yield of 1.87% per EMB with only 1.39% of rsEMB detecting clinically silent acute rejection [5]. Accordingly, Shah et al. reported a diagnostic yield of rsEMB of approx. 3% in asymptomatic patients in the first 6 months after HTx and of near 0% in the following 6 months [7]. Whereas Hamour et al. conducted approximately 14 rsEMB and Shah et al. conducted approximately 11 rsEMB per patient in the first year after HTx, the frequency of rsEMB at our centre in the first year after HTx has been significantly lower over the past decade. Yet, the efficacy and safety of lower frequency rsEMB has not been investigated so far.

Therefore, we thought to investigate rsEMB results, incidence of rejections requiring treatment, rate of unscheduled symptom-triggered EMB (stEMB), periprocedural complications, risk factors for rejection and outcome (including incidence of cardiac allograft vasculopathy (CAV) and mortality) of patients transplanted at our centre between 2004 and 2014.

## Methods

### Patients and data collection

At our institution, rsEMB were conducted monthly for the first 6 months with the first rsEMB 1 month after HTx and subsequently at month 9 and 12. Until 2006/2007, rsEMB were additionally conducted at month 8 and month 10 and until 2008/2009, rsEMB were conducted additionally 1 week after HTx. Evaluation for cardiac allograft vasculopathy (CAV), if feasible, was conducted once every year with coronary angiography in alternation with non-invasive testing including stress echocardiography, myocardial scintigraphy and CT angiography at the discretion of the attending physician. Clinical data of patients were obtained from medical records. The study was conducted according to the Declaration of Helsinki and approved by the local ethics committee of the medical faculty of LMU Munich. None of the transplant donors were from a vulnerable population. Data was accessed anonymously and the Institutional Review Board of our faculty waived the need for consent.

### Immunosuppression and target level surveillance

Immunosuppression was conducted using the combinations and target levels listed in Table 1. Additionally, oral prednisolone therapy was continued for the first 6 months, generally at a maintenance dosage of 5–7.5 mg/day. The distribution of immunosuppression combinations is quantified in Table 2. Target levels were evaluated every week in the first 6 months and every other week during months 6–12. The dosage of the immunosuppression was adjusted immediately via phone call in case of out-of-range plasma levels.

**Table 1:** Immunosuppression combinations and target levels.

period after HTx	TAC/MMF		SIR/MMF		EVE/MMF		TAC/SIR		TAC/EVE	
**Month 1–3**	10–12	1.5–4	12–15	1.5–4	10–12	1.5–4	5–8	5–8	5–8	5–8
**Month 4–12**	8–10	1.5–4	10–12	1.5–4	8–10	1.5–4	5–8	5–8	5–8	5–8

TAC = tacrolimus; MMF = mycophenolate mofetil; SIR = sirolimus; EVE = everolimus, plasma concentrations in ng/ml

**Table 2:** Patient characteristics.

Characteristics	All patients n = 282	Patients with rsEMB n = 218
	number (%)*	
**Age > 50 years**	159 (56)	118 (54)
**Female gender**	54 (19)	40 (18)
**Entity**		
ICM	103 (37)	75 (34)
DCM	144 (51)	116 (53)
other	35 (12)	27 (12)
**Gender mismatch**		57 (26)
M→F	9 (3)	7 (3)
F→M	70 (25)	50 (23)
**Initial treatment**		
TAC/MMF	230 (82)	187 (86)
TAC/m-TOR	27 (10)	27 (12)
CNI free	3 (1)	3 (1)
TAC/AZA	1 (0)	1 (0)
TAC	1 (0)	0
Unkown/not applicable	20 (7)	0
**Maintenance therapy changed within the first year**		
No		164 (75)
Yes		54 (25)
**Assist device**	66 (23)	43 (20)
**CMV status**		
Neg→neg	62 (22)	47 (22)
Pos→pos	66 (23)	52 (24)
Neg→pos	74 (26)	55 (25)
Pos→neg	80 (28)	64 (29)
**Ischemic time > 240 min.**	128 (45)	99 (45)
**Post-operativ ICU time > 5 days**		133 (61)
**HTx in 2011 or later**	86 (31)	63 (29)

Absolute values with percentages in parentheses.

* Deviation from 100% due to rounding. m-TOR implicating either sirolimus or everolimus.

### Definitions

Based on the guidelines of the *International Society of Heart and Lung Transplantation* (ISHLT 2010) for the care of heart transplant recipients [3], patients with symptomatic acute cellular rejection grade 1R or patients with rejection ≥ grade 2R on rsEMB independent of clinical symptoms were defined as requiring treatment with GC pulse therapy (Class I, Level C). Asymptomatic 1R acute cellular rejections diagnosed at rsEMB were generally not treated (Class I, Level C). Pathological grades of EMB were based on the 2004 ISHLT scoring system [2]. Every unscheduled visit where patients complained of symptoms that finally led to EMB was defined as stEMB. Control EMB (n = 27) were defined as EMB conducted for re-evaluation after GC pulse therapy and are not subject to this study. Live threatening complications, those requiring surgery, or resulting in an immediate tricuspid valve damage were defined as major complications (see Table 3). CAV was defined as significant coronary artery stenosis ≥ 50% or distinct rarefaction.

**Table 3:** Complications of routine surveillance EMB.

Complications	rsEMB n = 1552	Patients n = 218
	*number*	
**Acute major complications**		
Ventricular perforation requiring surgical treatment	4	4
Hematoma by the access point with surgical treatment	1	1
Pseudoaneurysm	2	2
Arrhythmia with defibrillation	1	1
Arrhythmia treated with drugs	1	1
Tricuspid insufficiency grade 3 immediately after biopsy	1	1
**Acute minor complications**		
Accidental access of the A. carotis (treated with compression)	1	1
Hematoma by the access point without surgical treatment	2	2
Horner´s syndrome after injection of narcotics (spontaneous regression)	1	1
Pericardial effusion requiring further controls	10	10

### Statistical analysis

χ2 analysis was used to compare discrete variables. For statistical analysis and Kaplan-Meier curves SPSS statistics (version 21, IBM, Armonk, NY, USA) was used. For all analyses, a p-value less than 0.05 was considered significant.

## Results

### Patient characteristics

Between January 2004 and November 2014, a total of 282 patients were transplanted at our centre. 53 patients died before the first EMB and 11 patients were not investigated by rsEMB (see outcome section below). Indications for HTx were ischemic cardiomyopathy (ICM) in 103 (37%), dilated cardiomyopathy (DCM) in 144 (51%) and other causes of cardiomyopathy in 35 (12%) patients. In the cohort with rsEMB (n = 218), initial treatment regimen consisted of tacrolimus/mycophenolate mofetil (TAC/MMF) in 187 patients (86%), TAC/mammalian target of rapamycin inhibitor (mTOR inhibitor; TAC/mTOR) with a combination of TAC with either sirolimus (SIR) or everolimus (EVE) in 27 patients (12%), calcineurin inhibitor-free immunosuppression (CNI-free, combination of SIR/MMF or EVE/MMF) in 3 patients (1%) and TAC/azathioprine (AZA) in 1 patient. The immunosuppressive regimen was maintained in 164 cases during the first year. In 54 patients the immunosuppression regimen was changed within the first year after HTx. In 4 of these patients treatment change was followed by an episode of acute rejection. 57 (26%) patients received an allograft from a donor with non-matching gender. 43 (20%) patients were transplanted after previous assist device implantation. The complete patient characteristics are shown in Table 2.

### Results of routine surveillance and unscheduled symptom-triggered EMB

**Fig 1:**
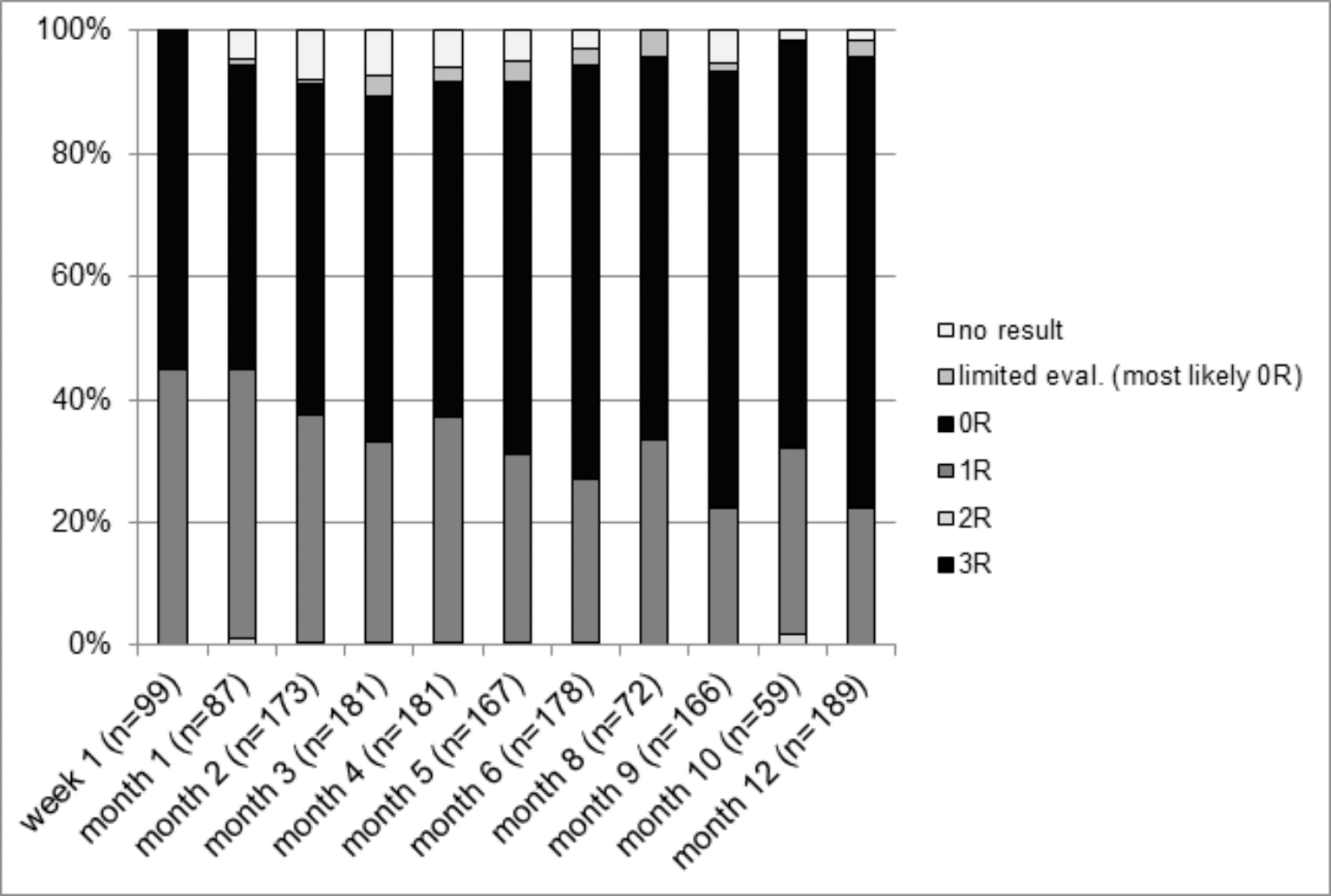
rsEMB results. Results of all rsEMB performed between 2004 and 2014 classified by pathological grading and time point.

Between 2004 and 2014, 1552 rsEMB were conducted in 218 patients (mean 7.1±2.5 rsEMB per patient) within the first year after HTx. EMB were performed via a femoral vein approach in 575 of cases and via a jugular vein approach in 922 of cases. The access could not be verified retrospectively in 86 cases. Pathological analysis of rsEMB revealed grade 0R in 948 (61%), 1R in 485 (31%), 2R in 6 (0.4%) and 3R in 1 cases. 36 rsEMB (2.3%) showed limited evaluability as, in the majority of cases, they consisted of only 1 specimen. 76 rsEMB (4.9%) could not be graded as they did not yield any evaluable heart tissue samples. The distribution of the grades of rejection between the various time points is illustrated in Fig 1. The 7 rejections ≥ grade 2R occurred in 7 asymptomatic patients (incidence of asymptomatic acute rejection 3.2%). Further, 9 rejections grade 1R required treatment based on rsEMB results in combination with symptoms (incidence of symptomatic acute rejection 4.1%). Altogether, 16 patients (7.3%) suffered from rejection requiring treatment diagnosed during routine follow-up (rsEMB). Although more acute rejections occurred in the first 6 months, we did not observe a significant difference in the rate of rsEMB revealing rejection between the first 6 months and the following 6 months (1.13% of rsEMB in month 1–6 vs. 0.87% of rsEMB in month 7–12; p = 0.58). During 1-year follow-up only 6 unscheduled stEMB were conducted in 6 different patients and resulted in the detection of 1 grade 0R and 5 grade 1R rejections. Clinical symptoms in 3 of these patients were worsening of general condition, new T-negativation in the ECG or septic shock. In the remaining 3 patients, the reason for the unscheduled stEMB could not be defined. Retrospectively, we found that only 2 patients with grade 1R rejection detected by stEMB underwent GC pulse therapy. Incidence of hospitalization for symptomatic acute rejection diagnosed by either rsEMB or unscheduled stEMB was 5.1% in the first year after HTx. The total incidence of hospitalization for rejection treatment (asymptomatic and symptomatic rejections, diagnosed by rsEMB or stEMB) was 8.3% during the first year after HTx.

### Complications of routine surveillance and symptom-triggered EMB

Of 1552 rsEMB, 10 (0.6%) led to major and 14 (0.9%) to minor complications. stEMB could be performed without any complications. Table 3 lists acute major and minor complications in detail. The leading major complication was ventricular perforation requiring surgical treatment (0.3%, EMB performed via a femoral approach in 2 patients) followed by pseudoaneurysm at puncture site (0.1%) and arrhythmia with the necessity of defibrillation (ventricular fibrillation, 1 patient) or additional drug treatment (atrial flutter, therapy with verapamil in 1 patient).

### Outcome

**Fig 2:**
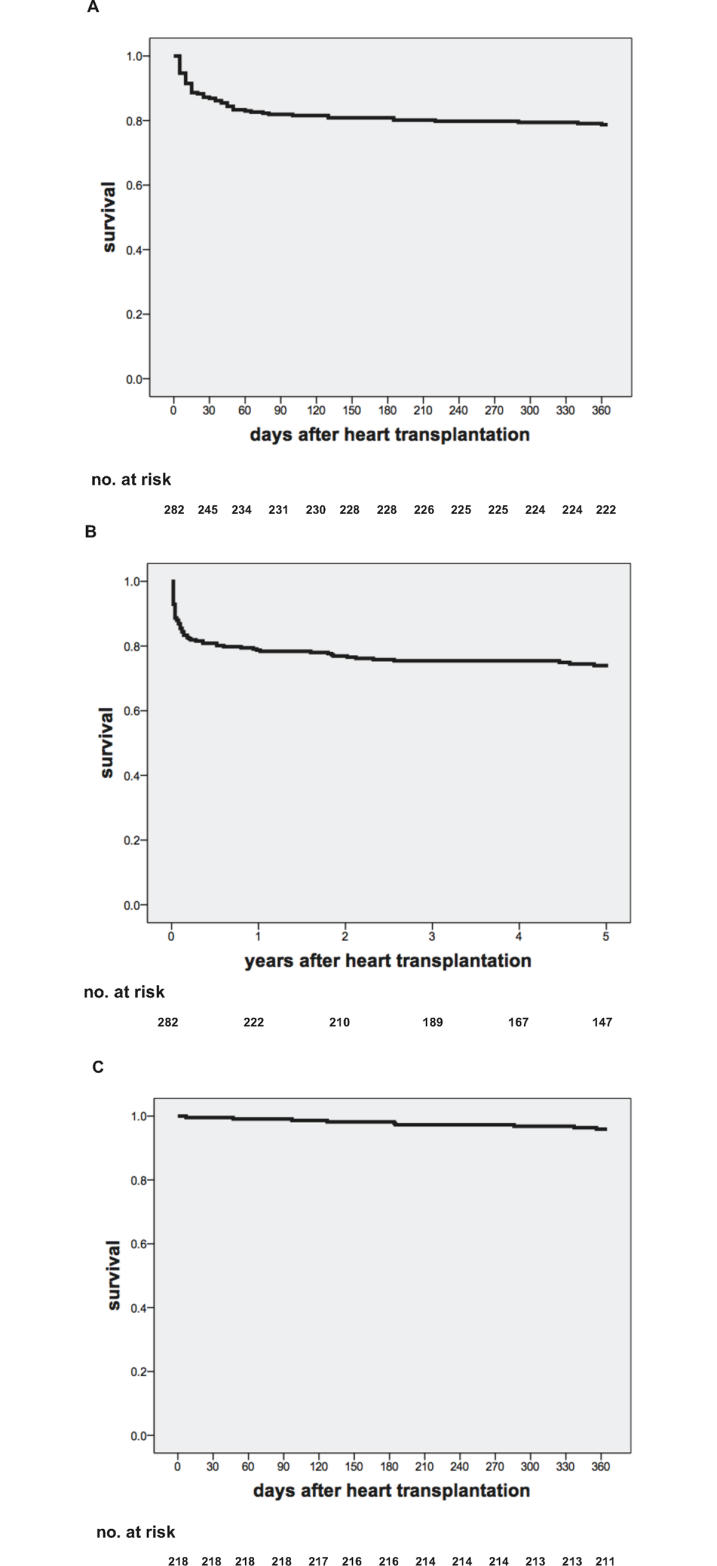
Kaplan-Meier survival curves. (A) 1-year survival (78.7%) and (B) 5-year survival (74%) of all 282 patients transplanted between 2004 and 2014. (C) 1-year survival (96.6%) of the cohort investigated by rsEMB (218 patients).

Survival at 1 year was 78.7% for all 282 patients who underwent heart transplantation. Long-term follow-up for a median of 5.2 ± 3.7 years showed a 5-year survival of 74% (Fig 2A and 2B). Of the 64 patients not followed up with neither rsEMB nor stEMB, 53 patients died before the first EMB. 11 patients survived at least until 1-year follow-up without any EMB follow-up. Of the 218 patients diagnosed with rsEMB, survival at 1 year was 96.8% (Fig 2C) and 5-year survival was 91% (data not shown). Causes of death as well as median survival of the patients who died in the two cohorts are shown in Table 4. The majority of deaths was attributed to sepsis (22 patients (7.8%) in total), immediate perioperative mortality (12 patients (4.3%) in total) and right heart failure (10 patients (3.6%) in total). In the cohort which died prior to the first EMB, 2 patients died of ventricular fibrillation, 1 patient of an unknown cause and 2 patients died of graft failure. One patient of the latter underwent autopsy and showed no cellular infiltrations as signs of acute cellular rejection. The other 4 patients did not undergo autopsy and therefore, in these 4 patients acute rejection could not be ruled out as a potential underlying cause of death.

**Table 4:** Causes of death in the two cohorts.

Characteristics	Deaths	Survival in days
	*number*, number (%)* *or median [IQR]*	
**Cohort without rsEMB (n = 64)**	53 (83)	10 [4–35]^#^
**Cause of death (1-year follow-up)**		
Cerebral hemorrhage/infarction	3 (5)	5, 12, 28
Perioperative death	12 (19)	1 [0.75–1]
Ventricular fibrillation	2 (3)	7, 43
Sepsis	19 (30)	35 [11–54]
Right heart failure	10 (16)	10 [9–20.5]
Multiple organ failure	3 (5)	4, 10, 20
Graft failure	2 (3)	14, 42
Unkown	1	64
HIT	1	9
**Cohort with rsEMB (n = 218)**	7 (3)	185 [155–311]
**Cause of death (1-year follow-up)**		
Sepsis	3	126, 184, 336
Malignancy	1	183
Fatal Bleeding	1	96
Sudden cardiac death	2	285, 355

* Deviation from 100% due to rounding.

# Median survival of the 53 patients who did not survive until the first EMB.

In the rsEMB cohort, 2 patients died of sudden cardiac death, 1 after a prior rejection grade 1R requiring treatment and the other after a prior rejection grade 1R which was not treated. Again, no autopsy was performed of these 2 patients. In total, in 6 patients’ deaths (2.1%), acute rejection could not be ruled out as a potential underlying cause. CAV was evaluated invasively by coronary angiography in 176 patients at 1-year follow-up, 145 patients at 2-year follow-up and 32 patients at 3-year follow-up. Non-invasive evaluation by CT angiography, myocardial scintigraphy or stress echocardiography was conducted in 1 patient at 1-year follow-up, 29 patients at 2-year follow-up and 32 patients at 3-year follow-up. The cumulative incidence of CAV at 3-year follow-up was 17%.

### Risk factors for acute cellular rejections

**Fig 3:**
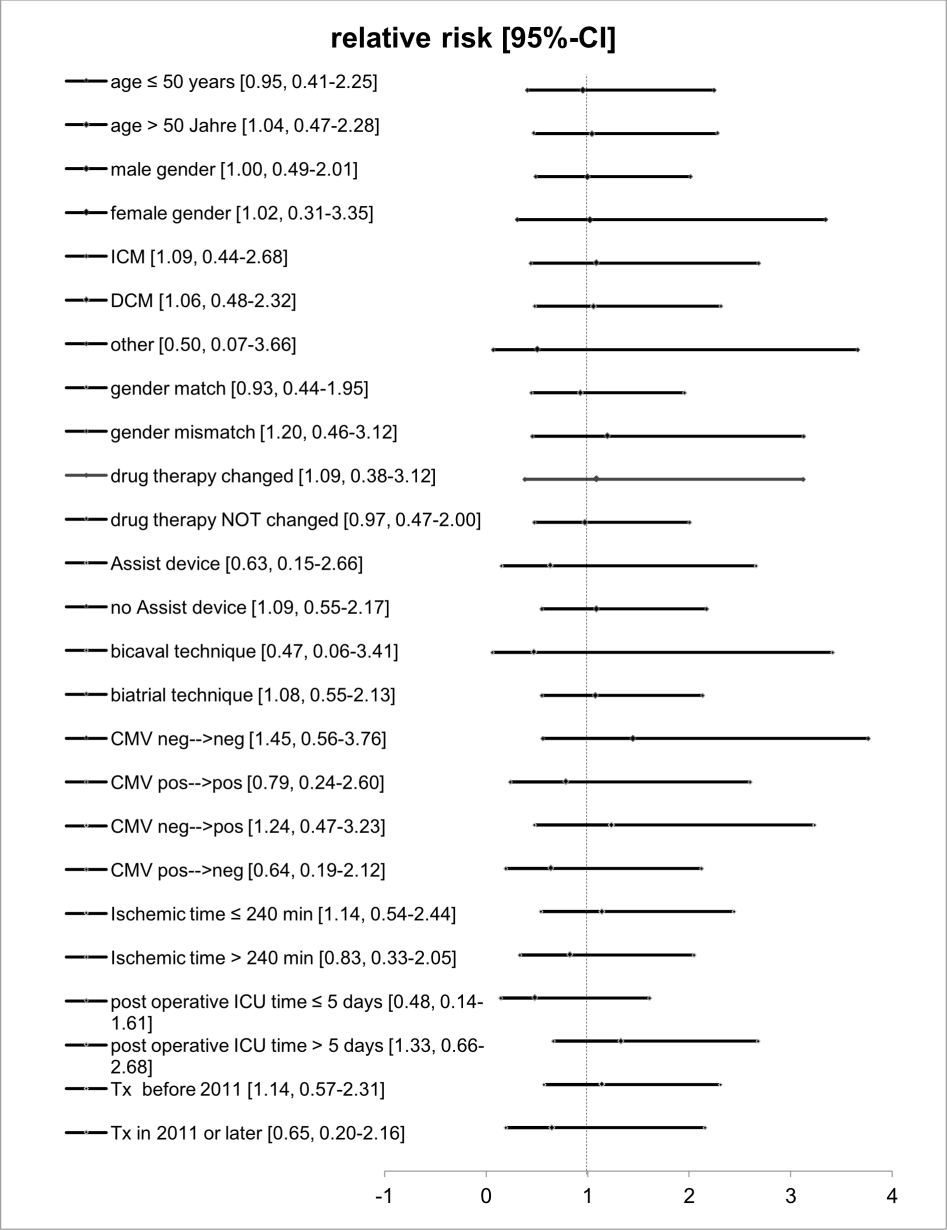
Risk factors for acute allograft rejection. Subgroup analyses were conducted to identify cohorts at higher risk for acute allograft rejection. None of the subgroups investigated significantly differed from the total cohort regarding probability of rejection. CI = confidence interval.

To investigate whether age, etiology of heart failure, gender mismatch, immunosuppression and change of immunosuppression, transplantation after previous assist device implantation, cytomegalovirus (CMV) status, transplant ischemia time, postoperative ICU time or the era, when transplantation was undergone, would affect the risk for acute cellular allograft rejection we performed subgroup analyses. None of the analyzed subgroups revealed a significantly higher rejection rate compared to the entire cohort (Fig 3).

## Discussion

In this retrospective, single centre, observational study we evaluated 1552 rsEMB of 218 patients in the first year after HTx between 2004 and 2014. The incidence for rejections requiring treatment diagnosed by rsEMB was 7.3%; 3.2% of patients had an asymptomatic rejection ≥ grade 2R. Despite a lower frequency of rsEMB (mean 7.1 ± 2.5 rsEMB per patient) only 6 patients underwent unscheduled stEMB with 2 patients revealing rejections requiring treatment. Together, this results in an overall rejection incidence of 8.3%. After 1-year follow-up, 6 deaths (2.1%) could be possibly attributed to acute rejection. Only 0.6% of the rsEMB resulted in major complications. Incidence of CAV was 17% at 3-year follow-up. Furthermore, we could not identify risk factors for acute cellular rejection.

To the best of our knowledge, this is the first centre report which evaluated the incidence of moderate ≥ grade 2R as well as grade 1R rejections requiring treatment according ISHLT guidelines [3]. The guideline authors recommend treatment of symptomatic grade 1R rejections and of ≥ grade 2R rejections regardless of symptoms. Two other recently published studies on the diagnostic yield of rsEMB by Hamour et al. and Shah et al. solely evaluated the incidence of ≥ grade 2R rejections. Furthermore, the ISHLT registry did not distinguish hospitalizations due to symptomatic grade 1R from those caused by ≥ grade 2R rejections [5, 7, 8]. We show that the incidence of symptomatic grade 1R rejections diagnosed with either rsEMB or with unscheduled stEMB was higher than that of ≥ grade 2R rejections requiring treatment (11 for all grade 1R rejections requiring treatment vs. 7 for ≥ grade 2R rejections).

With 8.3% of patients hospitalized for rejection treatment after rsEMB or stEMB during the first year after HTx, our centre detected less clinically significant rejections then others. For example, Shah and colleagues described an incidence of 23.3% in the cohort treated with modern immunosuppressive regimen and transplanted between 2000 and 2011 [7]. Kobashigawa and colleagues reported an incidence of acute rejections ≥ grade 3A or hemodynamic compromise rejection requiring treatment of 23–36% in a randomized trial with 343 patients [9]. In the twenty-eighth report of the registry of the ISHLT, the incidence of hospitalization for acute allograft rejection (humoral or cellular) was 26% for patients transplanted between 2001 and 2009 [8]. Lately, the ISHLT registry report of 2014 –with an incidence of treated rejections (cellular and humoral) of 13%—showed a more comparable incidence during the first year after HTx. Nevertheless, the reason for the lower incidence of rejections in our cohort remains unclear and it cannot be ruled out that lower frequency rsEMB are simply not able to detect all episodes of acute rejection.

To address the clinical impact of possibly undetected episodes of acute rejection we additionally evaluated the total amount of unscheduled stEMB, the mid-term incidence of CAV and mortality at various time points including causes of death. We could show that the relatively low frequency of rsEMB at our centre did not automatically lead to frequent unscheduled hospitalizations due to rejections as we only observed 6 stEMB during the first year after HTx. CAV is one of the most common causes for long-term mortality. Besides non-immunological damage such as ischemia reperfusion injury, infection and hypertension, immune-mediated injury due to antibody-mediated and cellular rejection can lead to CAV [10]. Invasive and non-invasive evaluation showed an incidence of CAV of 17%, which is comparable with incidences evaluated in a large international registry: Stehlik and colleagues reported an incidence of 20% at 3-year follow-up in patients transplanted between 2001 and 2009.[8] Survival of the cohort investigated by rsEMB was 96.6% at 1-year follow-up. We found a death rate of maximally 2.1% potentially attributable to acute rejection in the first year after HTx, which is comparable to death rates attributed to acute rejection observed within the ISHLT registry [8]. Although we did not follow up our patients with rsEMB beyond 1 year after HTx, our long-term survival data is internationally comparable [8].

As EMB can lead to significant morbidity due to severe complications the evaluation of such complications is essential to determine the net benefit for the patients. Whereas incidences for perforation/pericardial tamponade, myocardial infarction or substantial bleeding at puncture side are as low as approximately 0.5–2.0%, [5, 11–13] damage of the tricuspid valve can cause severe regurgitation in up to 14% of cases eventually leading to relevant morbidity [14]. In our cohort, incidences of acute complications were low and comparable with those from the literature as we could observe major complications in only 0.6% of rsEMB. Especially, severe EMB-related tricuspid valve damage only appeared once, which is in line with more recent findings by Fiorelli et al., who observed acute damage of the tricuspid valve after EMB in only 2 of 417 patients [15].

Interestingly, age, gender, cause for heart failure, gender mismatch, immunosuppression therapy or change of maintenance therapy during the first year, CMV status, ischemic time of the transplant, postoperative ICU time or transplantation before 2011, did not affect susceptibility for allograft rejection in our study. These findings are in contrast to a multi-institutional study from 1994 [16]. In this study, the investigators found that female gender of recipient or donor correlated with an increased risk for acute allograft rejection. However, the number of allograft rejections in our study was probably too low to detect differences in our patient population. Consistent with other single centre studies and registry data [5–7],

We found a decrease of acute rejections over time with the majority of rejections requiring treatment taking place in the first 6 months after HTx. However, these data may be confounded by i) the relatively low number of patients when compared to larger registry data and ii) by the fact that rsEMB frequency was simply higher in the first 6 months.

In this single-centre, observational study we retrospectively evaluated incidences of acute rejection, complications, incidence of CAV and survival in a cohort investigated by lower frequency rsEMB compared to other studies. Lower frequency of rsEMB does not seem to be automatically associated with higher incidences of unscheduled symptom-triggered hospitalizations, higher incidence of CAV or higher mortality due to acute rejection when compared to large registry data. On the other hand, the rate of major complications was as low as 0.6%.

Due to the lack of a control cohort, the limited number of patients and the retrospective design, no direct conclusions can be drawn from our observations so far. Larger prospective trials are warranted to address the gap of knowledge on the optimal frequency for rsEMB.
